# Unraveling the Decomposition Pathways of LaS-TaS_2_ Misfit-Layered Compound Nanostructures under Extreme Electrical Currents by In Situ TEM

**DOI:** 10.1021/acs.jpcc.5c03498

**Published:** 2025-07-21

**Authors:** Simon Hettler, MB Sreedhara, Reshef Tenne, Raul Arenal

**Affiliations:** † Instituto de Nanociencia y Materiales de Aragon (INMA), CSIC-Universidad de Zaragoza, 50009 Zaragoza, Spain; ‡ Laboratorio de Microscopías Avanzadas (LMA), 16765 Universidad de Zaragoza, 50018 Zaragoza, Spain; § Solid State and Structural Chemistry Unit, 29120 Indian Institute of Science, Bengaluru, Karnataka 560012, India; ∥ Department of Molecular Chemistry and Materials Science, 34976 Weizmann Institute of Science, Rehovot 7610001, Israel; ⊥ ARAID Foundation, 50018 Zaragoza, Spain

## Abstract

Nanostructures of the misfit-layered compound (MLC) LaS-TaS_2_ are brought to breakdown by application of high electrical currents within a transmission electron microscope. Imaging, diffraction, and spectroscopy techniques are employed to study their decomposition process and the resulting structures. The main decomposition route is the breakdown of the TaS_2_ layers, which induces the formation of metallic Ta on the surface of the nanostructures when the critical current density is surpassed. This observation confirms the assumption that TaS_2_ is the current-carrying layer in these types of MLCs. The different behavior of tubular 1D and 2D structures is revealed, and a metallic glass phase made of La, Ta, and S could be observed upon exceeding a threshold current. The work shows how in situ transmission electron microscopy can help understand the breakdown mechanism of electrical devices or connectors.

## Graphical abstract

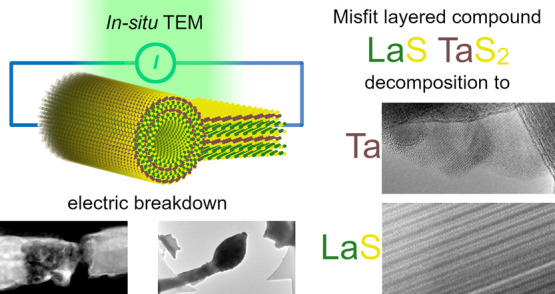

## Introduction

Electronic conduction is a complex phenomenon that depends mainly on the electronic band structure of materials, but can be strongly influenced by defects such as grain boundaries or geometrical constraints, for example, in nanoscale electronic devices.^1–8^ Considerable research efforts have been dedicated to elucidate these dependencies within nanodevices and nanostructures using in situ approaches based on electron microscopy.^9–14^ For example, the contribution of grain boundaries to the conductivity of Cu was quantitatively determined by in situ scanning electron microscopy (SEM),^15^ and the impact of stacking faults on the stability toward electromigration at high electrical currents in Ag nanowires was revealed by in situ transmission electron microscopy (TEM).^13^ TEM-based techniques have the advantage that the process can be followed at extremely high spatial resolution, but a clean sample must be prepared and good electrical contacts are crucial for obtaining valid information on the samples.^16^ An important field of research is the study of electromigration and Joule heating leading to the electrical breakdown of devices or (nano)­materials with the aim of improving their design and critical current density.^13,17–20^

A promising class of materials for applications in (thermo-)­electricity are misfit-layered compounds (MLCs), which consist of two layered materials alternately stacked on top of each other.^21,22^ Nanotubes (NTs) made of an MLC based on strontium, cobalt and oxygen have been shown to have an extraordinary high ampacity, that is, allowing high current densities.^20^ The two layers of MLCs typically consist of a distorted rock-salt unit MX and a hexagonal unit TX_2_, with metals M, transition metals T and chalcogens X (see sketch in Figure 1a,b). Recently, NTs made of sulfur-based MLCs have been synthesized and investigated in large amounts and considerable variety.^23–27^ The electronic conduction in MLCs is assumed to be dominated by the TX_2_ layer, while the MX can typically be seen as an electrical insulator and a phonon glass,^28^ but direct experimental evidence for this assumption is still missing. The electrical properties and also the electric breakdown of several MLCs and related layered materials have been investigated before.^20,29–32^

**Figure 1:**
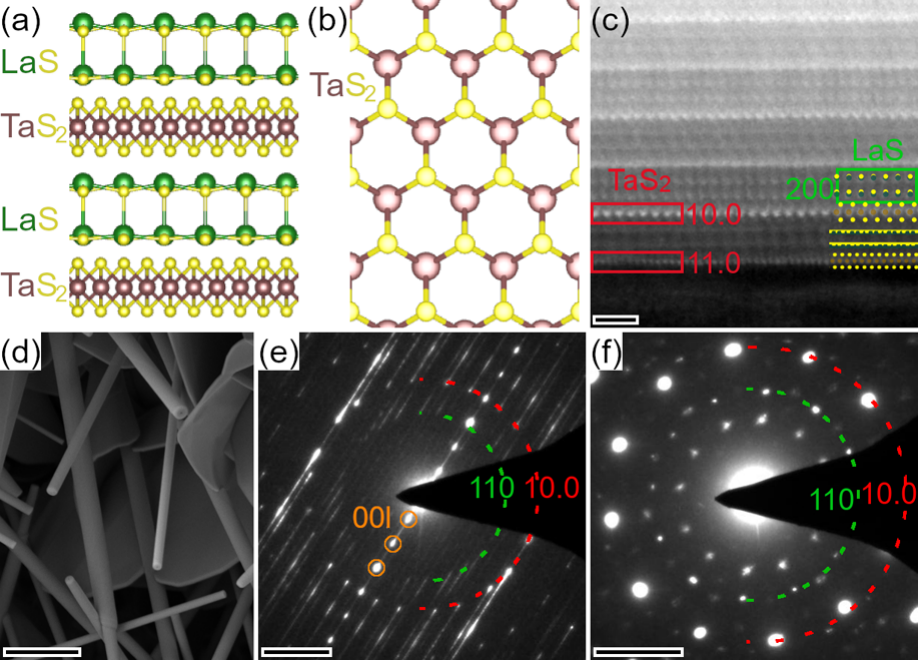
Structure of the MLC specimens used for the in situ experiments. (a) Sketch of a stack with alternating LaS and TaS_2_ layers along the *c*-axis of the MLC. (b) Sketch of hexagonal structure of TaS_2_ in the *a–b* plane, omitting the LaS. (c) STEM-HAADF image of the outer layers of a LaS-TaS_2_ NT with atomistic structure model overlaid and lattice distances of both subsystems marked. Reproduced from ref 33. Copyright 2020 American Chemical Society. (d) SEM image of the reaction product with mostly nanotubular structures and flakes. (e, f) SAED patterns of (e) a MLC NT and (f) a MLC flake. The 10.0 reflection of the TaS_2_ (red) and the 110 reflection of the LaS (green) sublattices have been indicated by dashed circles. The tubular structure causes a streaking of the reflections and leads to the presence of strong 00*l c*-axis reflections (orange), which are perpendicular to the NT axis. Scale bars are (c) 1 nm, (d) 2 μm and (e, f) 2 nm^–1^.

In this work, we investigated the behavior of NTs and flakes made of a LaS-TaS_2_ MLC^33^ under extreme electrical current densities by in situ TEM. This approach permitted studying the underlying complex physicochemical processes of the breakdown mechanism of the nanostructures at highest spatial resolution. The experiments indicate that the main current-induced breakdown mechanism of the MLC nanostructures is produced by the decomposition of the current-carrying TaS_2_ layer forming metallic Ta. In MLC flakes, deintercalation of LaS and bending of the flake was observed.

## Materials and Methods

### Microscopic Techniques

Two aberration-corrected Titan microscopes (Thermo Fisher Scientific), one image- and the other probe-corrected, were used for conducting the in situ experiments. High-resolution (HR)­TEM imaging and selected-area electron diffraction (SAED) were performed in the image-corrected microscope and scanning (S)­TEM using a high angle annular dark field (HAADF) detector combined with energy-dispersive X-ray spectroscopy (EDX) were performed in the probe-corrected microscope. A DENSsolutions Wildfire TEM sample holder was used in combination with custom chips, whose design is shown in the Supporting Information (SI), Section S1. Their fabrication is detailed elsewhere.^16^ Scanning electron microscopy (SEM) and focused-ion beam (FIB) processing were performed in a Helios 650 dual-beam instrument (Thermo Fisher Scientific).

Correlative Raman spectra were acquired from the specimens on the chip using a confocal Raman Alpha 300 M+ (WiTec) spectrometer with a 633 nm laser operated at a power below 1 mW and a 100× objective with a numerical aperture of 0.9. The spectrometer was operated using a grating with 1800 grooves per mm.

### Synthesis and Structure of the Misfit-Layered Compounds

We used NTs and flakes made of Y-doped (about 1 at %) LaS-TaS_2_ for the in situ experiments. For the sake of simplicity, the material will be denoted solely as LaS-TaS_2_ in the following. It is noted that all the studied nanomaterials however include this 1 at% Y doping. The NTs correspond to the “Y10” specimen of our recent work,^33^ where synthesis, structure and composition are described and discussed in detail. In short, the chemical vapor transport (CVT) technique was used to synthesize the NTs and flakes following a well-established protocol by mixing the precursors and a transport agent (TaCl_5_) in an agate mortar and sealing them in a quartz ampule under vacuum (<10^–5^ Torr). The MLC material is synthesized by annealing the ampule in a preheated vertical furnace in two steps with opposite temperature gradients between the bottom and top of the ampule. In the first step, the ampule is heated at 350 °C/800 °C for 1 h and subsequently at 857 °C/400 °C for 6 h. After cooling down to room temperature, the powder was collected from the ampule.

MLC nanostructures are a complex material and Figure 1 reviews the structure of the as-synthesized product used for the in situ experiment.^33^ The stack of alternating LaS and TaS_2_ layers along the *c*-axis of the MLC is sketched in Figure 1a. While the LaS exhibits a (distorted) cubic structure, the TaS_2_ crystallizes in a hexagonal structure (Figure 1b). The atomistic structure of the stack is revealed in the STEM-HAADF image taken at the border of a NT^33^ (Figure 1c), which allows identifying different crystallographic orientations. Predominantly tubular structures are visible in the SEM image of the product of the synthesis (Figure 1d). The SAED patterns taken from an individual NT (Figure 1e) and a flake (Figure 1f) show that both (*hk*0) and (00*l*) crystallographic directions are visible for the NT, while only planar reflections (*hk*0) are seen for a flake with its *c*-axis corresponding to the electron-beam direction.

### Sample Preparation

The as-synthesized powder was dispersed in ethyl alcohol by ultrasonication. A conventional TEM sample was prepared by drop-casting of the dispersion on carbon-coated commercial SiN_*x*_ grids with an array of holes with 2 μm diameters (PELCO). The sample was screened for potential candidates for the in situ experiments by TEM and their position within the hole array was identified in a low-magnification TEM image. Selected specimens were subsequently transferred to an in situ chip using a recently developed FIB-based specimen transfer technique.^16^ For this transfer, the region of interest together with a piece of the surrounding membrane is cut free and lifted out using a micro needle and focused-ion beam induced deposition (FIBID) of Pt. It is then placed between the two contacts of the microchip over a hole, previously milled by FIB. The membrane and nanomaterial are fixed and electrically contacted by an additional FIBID process with Pt. Finally, the membrane is milled away in the hole area. In this way, the nanomaterial remains the sole conductive path. This approach minimizes the damage and contamination to below a monolayer allowing to investigate the specimen in its pristine state.^16^ The preparation of the first specimen described in this article is documented in the SI, Section S2, where also a sketch of the process can be found.^16^

Although the transfer technique provides a good electrical contact,^16^ the tendency of the LaS-TaS_2_ MLC to oxidize caused minor surface oxidation of the nanomaterials resulting in non-Ohmic contacts. We therefore desist from calculating conductivities and instead focus on current densities.

### Application of Electrical Currents

A Keithley Instruments 2450 SourceMeter and a 2611A System SourceMeter (Tektronix) were used to apply DC electrical currents to the nanomaterial and to the on-chip heating element, respectively, while simultaneously measuring the voltage. Unless otherwise noted in the text, the high electrical current was applied to the nanomaterial in the form of relatively fast single sweeps of a duration of up to 1 min. Instead of applying sweeps with continuously increasing current, each sweep consisted of several steps with constant current and the period time of each step (typically 4 s) was set to coincide with the time step of the acquisition run of the (S)­TEM image series in parallel. In between these sweeps, the nanomaterials were characterized electrically at low currents in a static way to avoid Joule heating.

## Results and Discussion

We have performed several in situ experiments on the LaS-TaS_2_ nanostructures. Here, we present the results obtained from two main exemplary specimens, which consisted of a bundle of two NTs (first specimen) and a NT and flake (second specimen).

### First In Situ Specimen

Figure 2 shows TEM, SEM and STEM images of the first in situ specimen at different stages throughout the conducted experiment. The specimen consisted of a bundle of two LaS-TaS_2_ NTs with only one of the NTs spanning over the entire length of the hole as seen in the TEM image prior to the in situ specimen preparation, see Figure 2a. It is important to note the presence of a thin amorphous carbon (aC) film attached to the NTs, which is attributed to being caused by organic carbonaceous residues. An SEM image and a STEM image (inset) of the prepared in situ device are depicted in Figure 2b. More details of the preparation process are given in Section S2. The polarity of the applied electrical current and the resulting direction of the physical electron flow are indicated in Figure 2b. The following in situ experiment consisted of three main phases, which are briefly summarized here and whose results are described and discussed in detail within Figures 3 *–* [Fig fig5]:

- Phase 1: The annealing at moderate temperatures by an external heating element followed by the application of fast electrical current sweeps up to a maximum current of 325 μA leads to structural changes and an inhomogeneous rupture of the long NT (Figure 2c,d).
- Phase 2: The application of an additional fast electrical current sweep up to 300 μA causes the reconnection and decomposition of the central part of the NT followed by a second rupture of the NT (Figure 2e).
- Phase 3: The application of a constant electrical current with increasing intensity up to 140 μA first leads to a reconnection, then a further decomposition of the NT, and finally to the complete rupture of the NT and the aC film (Figure 2f).

**Figure 2:**
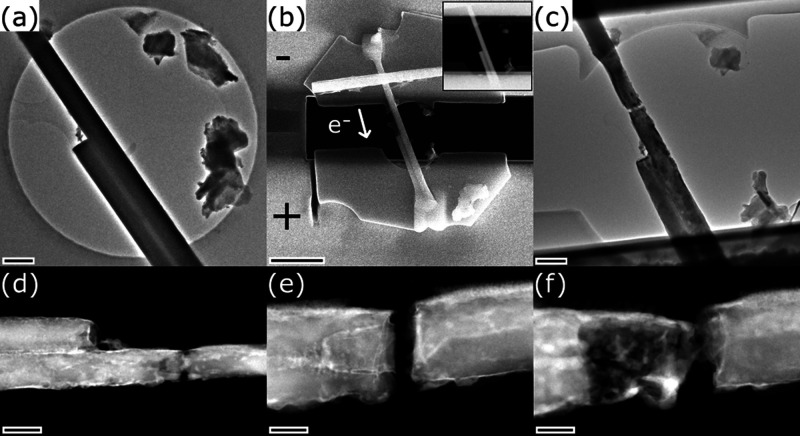
Overview of the first in situ specimen. (a) TEM image of the NT bundle suspended on a SiN_*x*_ membrane prior to in situ specimen preparation. (b) SEM image of the final device and STEM image (inset) of its central part. (c, d) TEM and STEM images of the ruptured NT after phase 1 revealing the cone-like shape of the rupture. (e) STEM image of the rupture after phase 2. (f) STEM image of the decomposed NT after the final phase 3. Scale bars are (a, c) 300 nm, (b) 2 μm, width of inset STEM image is 4 μm, (d) 200 nm, (e, f) 70 nm.

**Figure 3:**
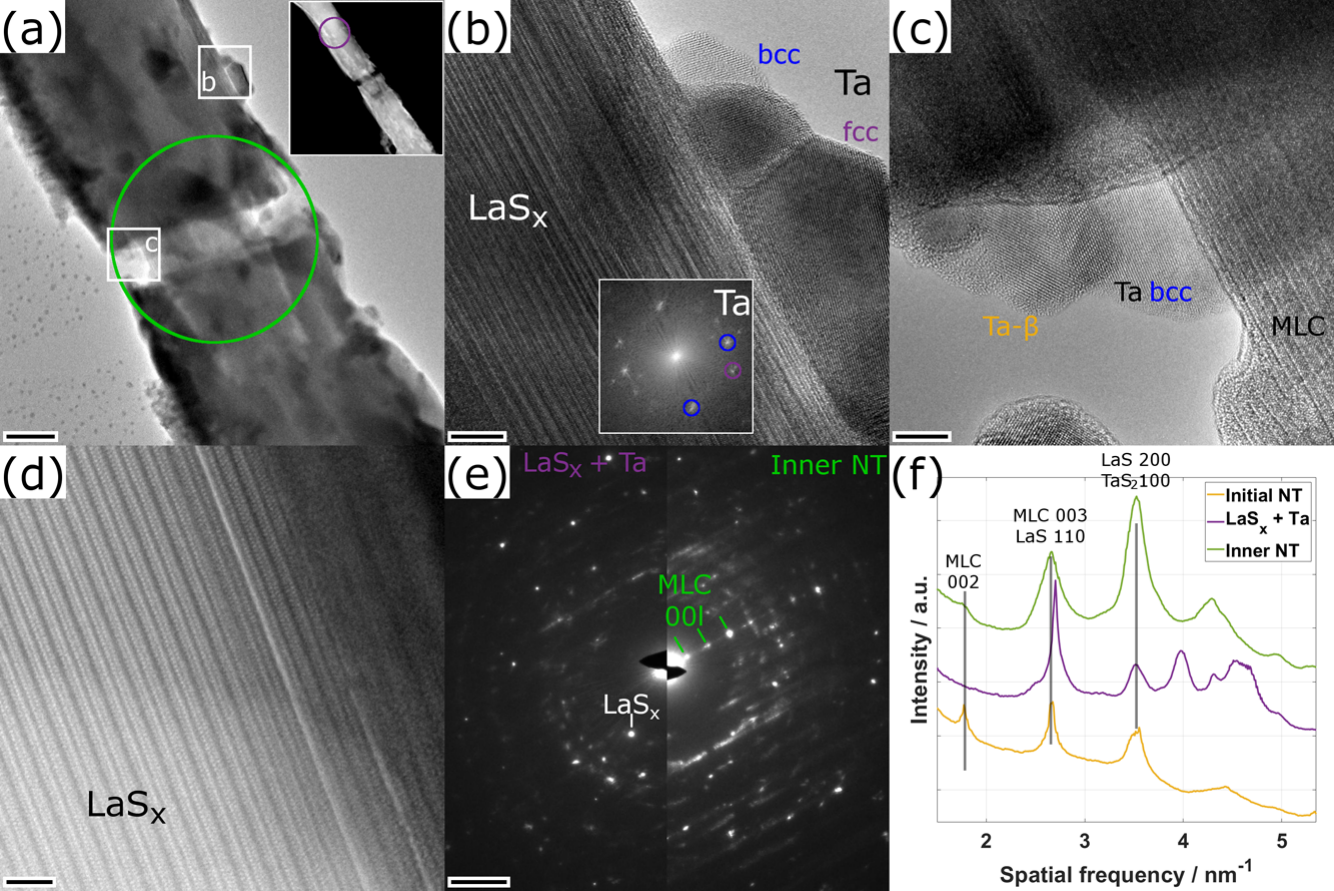
Analysis of the first specimen after the experiments of phase 1. (a) TEM and inset STEM image of the ruptured long NT. The areas for the SAED patterns displayed in (e) are marked by purple and green circles, respectively. The position of the HRTEM images shown in (b) and (c) are marked by white frames as indicated. (b) HRTEM image acquired from the protrusion grown on the NT edge as marked in (a). Inset power spectrum has been calculated by selecting the protrusions only. The [110] and [111] reflections of the bcc (blue) and fcc (purple) Ta lattice have been marked. (c) HRTEM image of the rupture site as marked in (a). The inner portion of the tube as well as several extracted Ta nanograins are seen. (d) STEM-HAADF image of the inner part of the NT close to the region shown in (b). (e) SAED patterns acquired from the upper NT and the ruptured site as marked in (a). (f) Radial profiles obtained from the SAED patterns in (e) compared to a profile obtained from the NT prior to the application of electrical currents. Scale bars are (a) 20 nm, width of inset STEM image is 1 μm, (b, d) 5 nm, inset power spectrum in (b) has width of 12 nm^–1^, (c) 2 nm, and (e) 2 nm^–1^.

The three phases are discussed in the following starting with

#### Phase 1

Prior to the application of electrical currents, the specimen was heated up to approximately 600 °C using the on-chip heating element. This step was performed with the intention to remove possible adsorbed contaminants without reaching temperatures sufficient to induce structural changes in the specimen. Subsequently, electrical current was applied in the form of gradually increasing sweeps up to a maximum current and followed by an immediate drop to zero applied current. The maximum current of the sweeps was increased stepwise. Supplementary Video S1 shows the evolution of the TEM images during a sweep up to a maximum current of 325 μA. Above a current of 275 μA, first a grainy contrast appears along the long NT, then the NT shrinks in diameter and protrusions appear at its edge. These currents correspond to current densities of 8.75–10 × 10^5^ Acm^–2^ considering a diameter of 200 nm of the single NT and neglecting the contribution of the aC film. Finally, the NT ruptures after the current drops to zero. Both TEM and STEM images taken after the rupture (Figure 3a) reveal that the NT adopts a cone-like shape at the rupture site, indicating that the inner and outer part of the NT ruptured at different positions. The images also confirm the presence of several protrusions at the edge, visible as darker areas in other regions of the NT.

Figure 3b shows a HRTEM image of the large protrusion marked in (a). The protrusion exhibits two crystalline nanograins as revealed in the power spectrum obtained by selecting only the protrusion area of the image. A STEM-EDX analysis, performed later using the probe-corrected microscope after air exposure, reveals that the protrusions are made of Ta, which is partially oxidized (S3). An analysis of the spots seen in the power spectrum in Figure 3b shows two slightly different lattice distances of 0.235 nm (marked by blue circles) and 0.21 nm (purple) linked to the upper and lower nanograins, respectively. The blue spots corresponding to the upper nanograin can be linked to the Ta bcc crystal structure ([111] = 0.234 nm, Crystallography Open Database (COD) #1541266 ^34^). The lower nanograin, however, does not fit to the bcc structure, but instead would agree with the fcc crystal structure of Ta ([200] = 0.211 nm, COD #1534932).^35^

Ta nanoparticles, which are a product of the MLC decomposition, are also found close to the rupture site (Figure 3c). The analysis of the corresponding power spectra reveals one grain of Ta with bcc structure and a second is found to crystallize as β-Ta (S4)^36^ (COD #2103245). The HRTEM images taken at the rupture site (Figures 3c and S5) show that the inner, cone-like part still possesses an intact MLC structure, however with a slightly larger *c*-axis periodicity of 1.16 nm (S5) compared to the reference value at room temperature of 1.15 nm.^33^ This lattice expansion indicates that the structure was quenched in an expanded state caused by the sudden drop to zero applied current, which stopped the Joule heating. The MLC structure in the inner cone at the rupture site is confirmed by SAED (right-hand side of Figure 3e). Its lattice expansion is visible from the shift of the 00*l* peaks toward smaller spatial frequencies (larger lattice spacings) compared to the pristine MLC NT (see SAED radial profiles in Figure 3f).

Figure 3d shows a STEM-HAADF image of the upper part of the NT taken close to the grown protrusions, revealing the loss of the MLC structure due to the removal of the TaS_2_ layers. The remaining structure exhibits a stacking periodicity of 1.26 nm made up of two slabs of LaS. The crystal structure could not be directly related to any known crystal structure based on La and S, but seems to be merely a modified form of the original MLC structure, with deintercalated TaS_2_ and in a slightly relaxed form. This deintercalated structure will be denoted as LaS_*x*_ in the following, owing to the possibility that a part of the S released upon deintercalation of TaS_2_ could have been absorbed by the LaS slabs.

The loss of the MLC structure throughout the entire NT in its upper part is confirmed by SAED (left-hand side of Figure 3e), where no 00*l* reflections of the MLC structure are observed and only several individual reflections can be seen. The main reflection at 0.37 nm, marked in Figure 3e, is associated with the LaS_*x*_ structure, since this value agrees well with the [201] lattice plane of La_2_S_3_ ^37^ (COD #1527122). The 0.37 nm lattice spacing is considerably different from the LaS [110] lattice spacing (0.41 nm), as visible from the SAED radial profiles in Figure 3f. A possible explanation is that, while Ta is fully extracted from the MLC in practically the entire upper NT caused by the high electrical current density, not all the released sulfur could leach out of the lattice. Presumably, the remaining sulfur from the inner part could react with the remaining LaS to form La_2_S_3_. The SAED pattern from the upper part (Figure 3e) reveals additional reflection spots with reduced intensity. An exact assignment of these reflections to a specific structure is difficult as both β-Ta and La_2_S_3_ structures exhibit a large number of reflections with similar lattice spacings.

The extraction of TaS_2_ from the MLC nanostructures is opposite to the main decomposition route observed by conventional high-temperature annealing. In this latter case, deintercalation of LaS layers from the MLC lattice was observed.^26,38^ This striking difference can be solely ascribed to the role of the electrical current, flowing exclusively through the conductive TaS_2_ layers of the MLC NTs and causing them to break down much earlier than the thermally induced deintercalation of LaS would occur. The observation of the pristine MLC structure in the inner part of the tube indicates that the current flow is higher in the outer layers closer to the surface of the NT. This observation is attributed to the sample geometry: The outer layers of the NT are in direct contact with the contact pad of the chip, causing the current to flow mostly through the outer layers. Furthermore, the insulating LaS slabs strongly limit the interlayer conduction, confining the current flow to the current-carrying TaS_2_ slabs at the surface of the MLC NT. The tubular structure together with in-plane conduction causes a distribution of the electrical current along the circumference of the NT as suggested by the appearance of extracted Ta all around the NT caused by electrical breakdown. The critical current density for the electrical breakdown of an individual layer will therefore be considerably higher than the value calculated for the entire NT.

#### Phase 2

After this first phase, the experiment was paused and continued in the probe-corrected microscope. A STEM-HAADF image of the specimen (Figure 4a) reveals that the aC film remained intact, guaranteeing an electrical contact. The electrical contact of the ruptured NT seems to be severely limited. The electrical current was then increased in a stepwise sweep up to a maximum current of 300 μA. Supplementary Video S2 shows the evolution of the NT and the underlying aC film during this increase and Figure 4b shows 5 images from this series. With increasing current, the specimen is expanding due to Joule heating and the gap between the two parts of the ruptured NT reduces in size. At the final stage of the applied current (300 μA), the two parts merge again and a bright line appears on the outside contour of the inner tube. The bright line can be linked to Ta, as it possesses a considerably higher atomic number (*Z* = 73) compared to La (57) and S (16) and thus causes an increased intensity of the STEM-HAADF signal. The leaching of Ta from the MLC in the outer part of the tube, which was observed in the first phase, is now seen in the inner NT, too. Here, again, the remaining inner tube was transformed into a LaS_*x*_ structure. This interpretation is confirmed by a STEM-EDX analysis and an additional HRSTEM image (S6).

**Figure 4:**
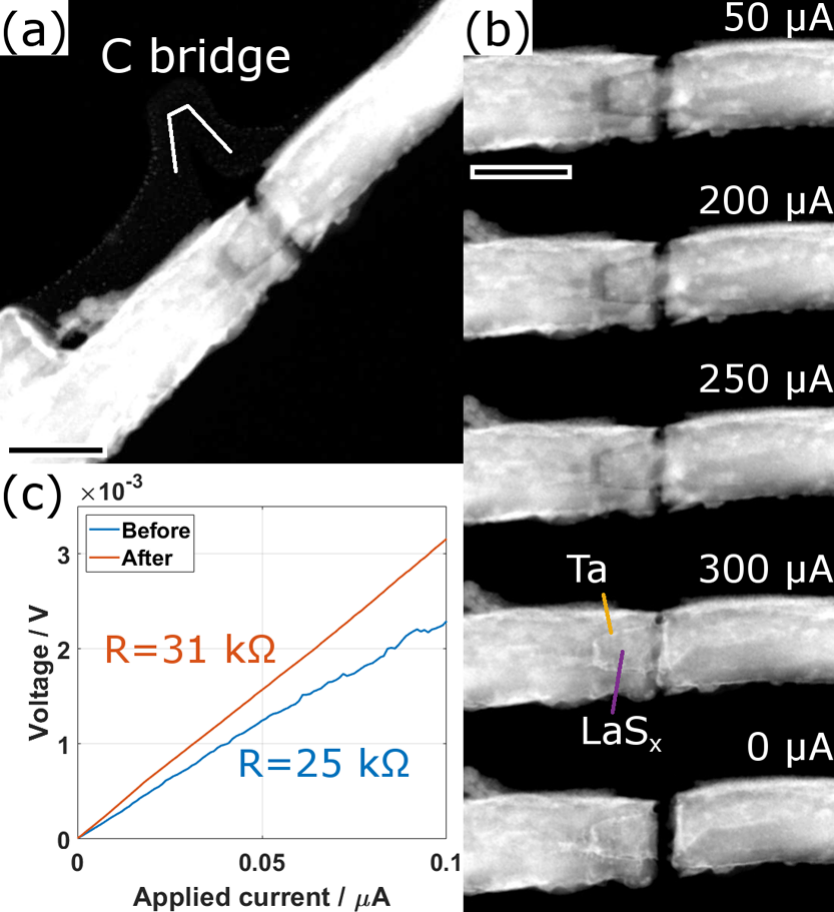
Analysis of the first specimen during and after phase 2. (a) STEM-HAADF image taken after the experiments of phase 1 reveals the cone-like rupture of the NT and the intact aC bridge. (b) Series of STEM images with increasing applied electrical current, as indicated. Identification of Ta and LaS_*x*_ was performed by STEM imaging and EDX as described in the text. (c) Comparison of the electrical characteristics before and after phase 2. Scale bars are 200 nm.

After turning off the applied current, the hot specimen cools rapidly, again causing a rupture of the NT, this time with a straight rupture line (Figure 4b). This indicates that the extracted Ta acts as a soldering agent that strongly connects the remainders of the inner and outer part of the NT, causing the NT to fracture at a different place. The aC bridge still remained intact (see Figure 5b). An electrical characterization at low currents reveals that the resistance slightly increased from 25 to 31 kΩ during the second phase (Figure 4c), indicating that likely there was a small contribution of the NT to the conductivity after phase 1. The considerably higher noise in the first measurement (blue curve) suggests that, as expected, this electrical contact was not very stable due to the rather loose contact between the two parts of the NT.

**Figure 5:**
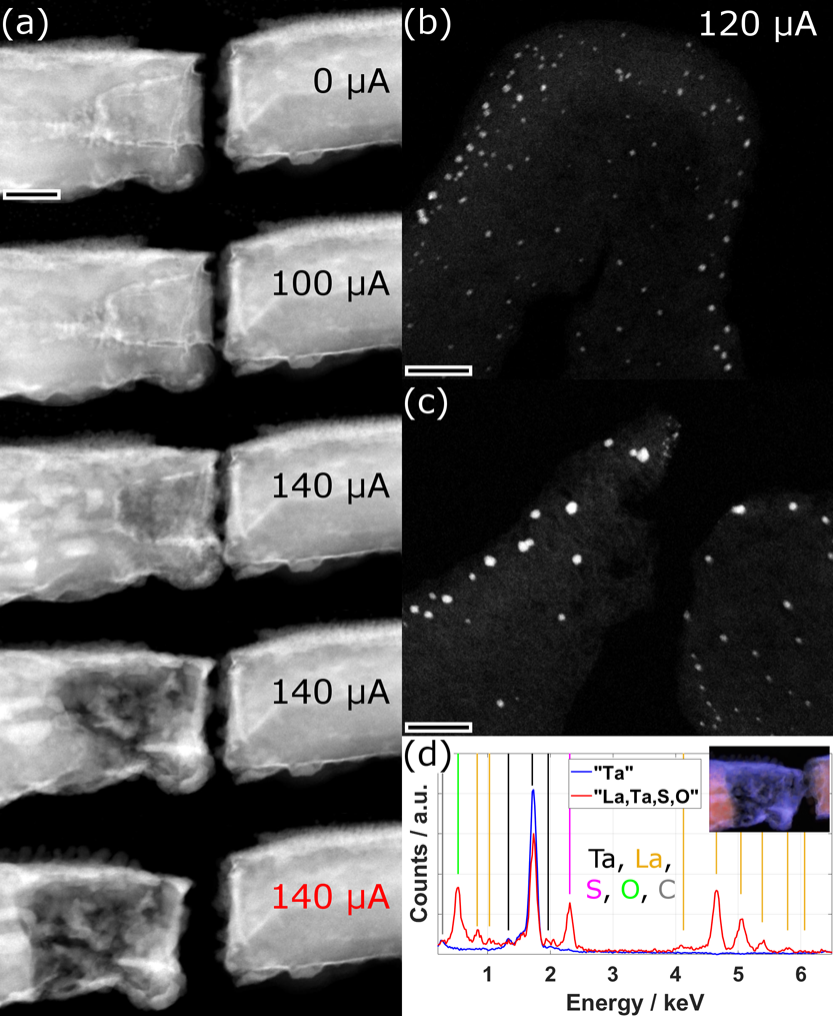
Analysis of the first specimen during and after phase 3. (a) Series of STEM-HAADF images of the central part of the ruptured NT at different applied currents. The final image was acquired after rupture without current flow. (b, c) STEM-HAADF images of the intact and ruptured aC bridge before and after phase 3, respectively. (d) EDX spectra attributed to two factors obtained from the statistical analysis denominated as “Ta” (blue) and “La,Ta,S,O” (red), respectively. Lines of the identified elements are indicated by different colors. The inset image shows the distribution of the two factors overlaid on the STEM-HAADF image. The EDX data was acquired at a tilt of the stage of 20°. Scale bars are (a) 70 nm and (b, c) 30 nm.

#### Phase 3

In the following third phase, a constant electrical current was applied, increasing manually to a maximum of 140 μA. The evolution of the STEM-HAADF image is shown in Figure 5a and in the Supplementary Video S3. Similarly to the previous phase, Joule heating of the specimen leads to its expansion until the two ruptured parts contact each other again at a current of 120 μA. In parallel, the NPs formed on the aC film start to diffuse out of the central zone due to the strong heat, which is similar to a process observed in our recent study.^39^ A strong modification of the specimen starts to occur once reaching a current of 140 μA. This modification manifests itself as a decrease in HAADF intensity of the NT, linked to the decomposition or removal of material from an area, approximately 200 nm long, in the left part of the NT. This process happens over the course of a few seconds before finally both the NT and the aC film break (Figure 5a,c), leading to a complete break of the electrical contact of the specimen.

An EDX analysis of the ruptured area of the NT reveals that the decomposed part is made entirely of Ta, while the remaining constituents of the specimen La, S, and O (from contact with air during microscope change) together with additional Ta, can be found only in the right and the outer left part of the NT. This is seen from the spectra and the composition map resulting from a statistical analysis of the acquired EDX map (Figure 5d). An SAED analysis of the decomposed part shows that the Ta exhibits a nanocrystalline structure with a majority of the grains crystallizing in fcc structure, but both bcc and β-Ta structure are also identified in the analyzed area (S7).

The results of the experiments conducted in phase 3 indicate that the physicochemical processes under electrical current flow are different for the original MLC compared to the deintercalated system composed of a mixture of metallic Ta and ceramic LaS_*x*_, found after phase 2 in the central part. The MLC is stable up to a certain current density at which point the TaS_2_ layers break down. This happens before the critical temperature is reached, at which the LaS would start to deintercalate. In contrast, the system with the metallic Ta (melting point of 3290 K) reaches considerably higher temperatures leading to the decomposition and diffusion of the La-based parts to colder areas and to the evaporation of S. A quantification of the entire EDX map of this area reveals a ratio between the main constituents of the MLC of Ta:La:S of 63:21:16 confirming the dominant presence of Ta.

### Second In Situ Specimen

The second in situ specimen consisted of a NT and a flake as seen from the TEM image shown in Figure 6a taken after in situ specimen preparation. The direction of the current is indicated in the figure by white arrows. For this specimen, the current feed is split into two parts on the top side. The NT and flake on the right side as well as the top left part of the flake are electrically connected to the contact pads of the chip. This configuration hinders the calculation of current densities. This specimen nevertheless serves to demonstrate current-induced physicochemical modifications of the MLC material not seen in the first specimen. In addition to the large flake and the NT, some smaller structures are visible. These structures are typically composed of the same elements (Ta, La, S) as the MLCs. The experiment was conducted in a similar way to the first specimen by applying a series of electrical current sweeps up to a maximum current of 414 μA. Three specific steps of the performed experiments, which provide additional information to those of the first in situ specimen, are presented in this section. Three TEM images are shown in Figure 6b–d illustrating the evolution of the specimen during the experiment. These include the melting and crystallization of a small flake; the different decomposition pathway of the flake compared to the NT and the breakdown of the device. These processes are detailed and discussed using Figures 7 and 8.

**Figure 6:**
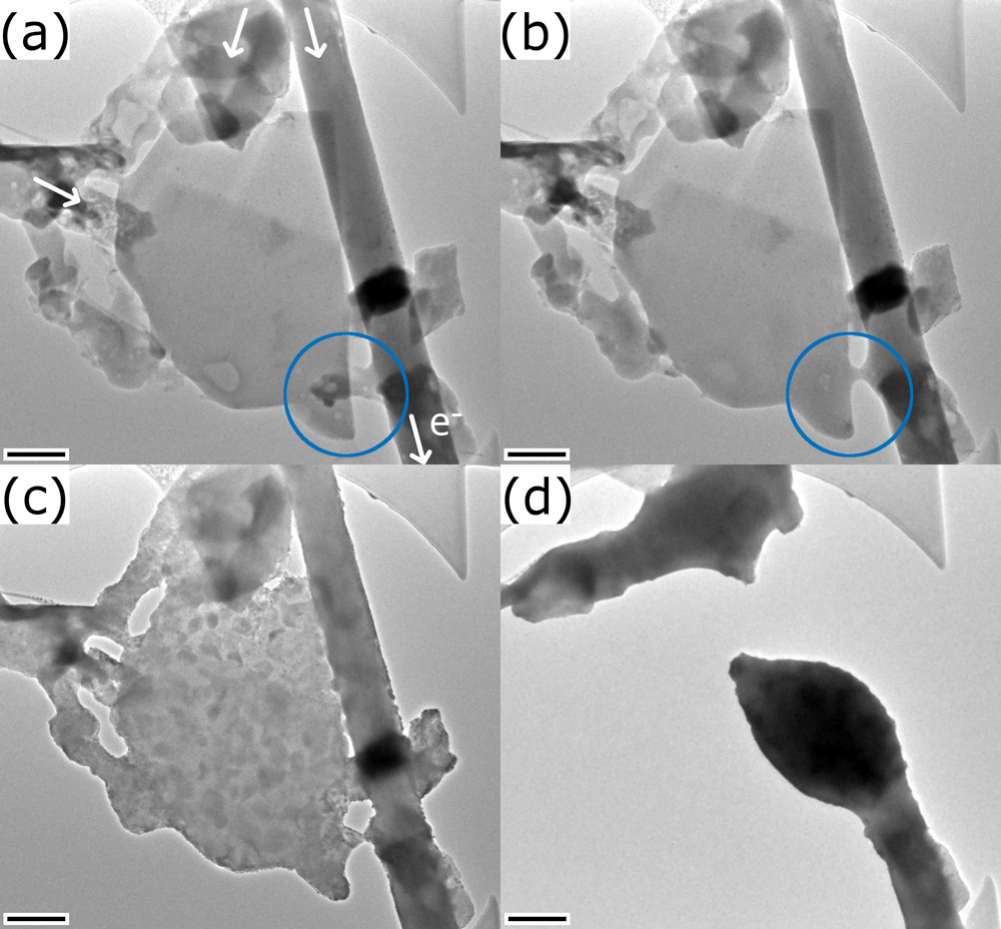
TEM images taken at four different times during the in situ experiments with the second specimen. (a) NT and flake after transfer to the in situ microchip. Direction and entry points for the electrical current have been indicated by white arrows. (b) The first observed structural modification corresponds to the melting of a small flake linking the large flake and the NT (marked by a blue circle). (c) At this stage, decomposition has started mostly in the flake, and protrusions have appeared on the edge of the NT as well. (d) Final rupture of the specimen leading to the formation of a droplet. Scale bars are 200 nm.

**Figure 7:**
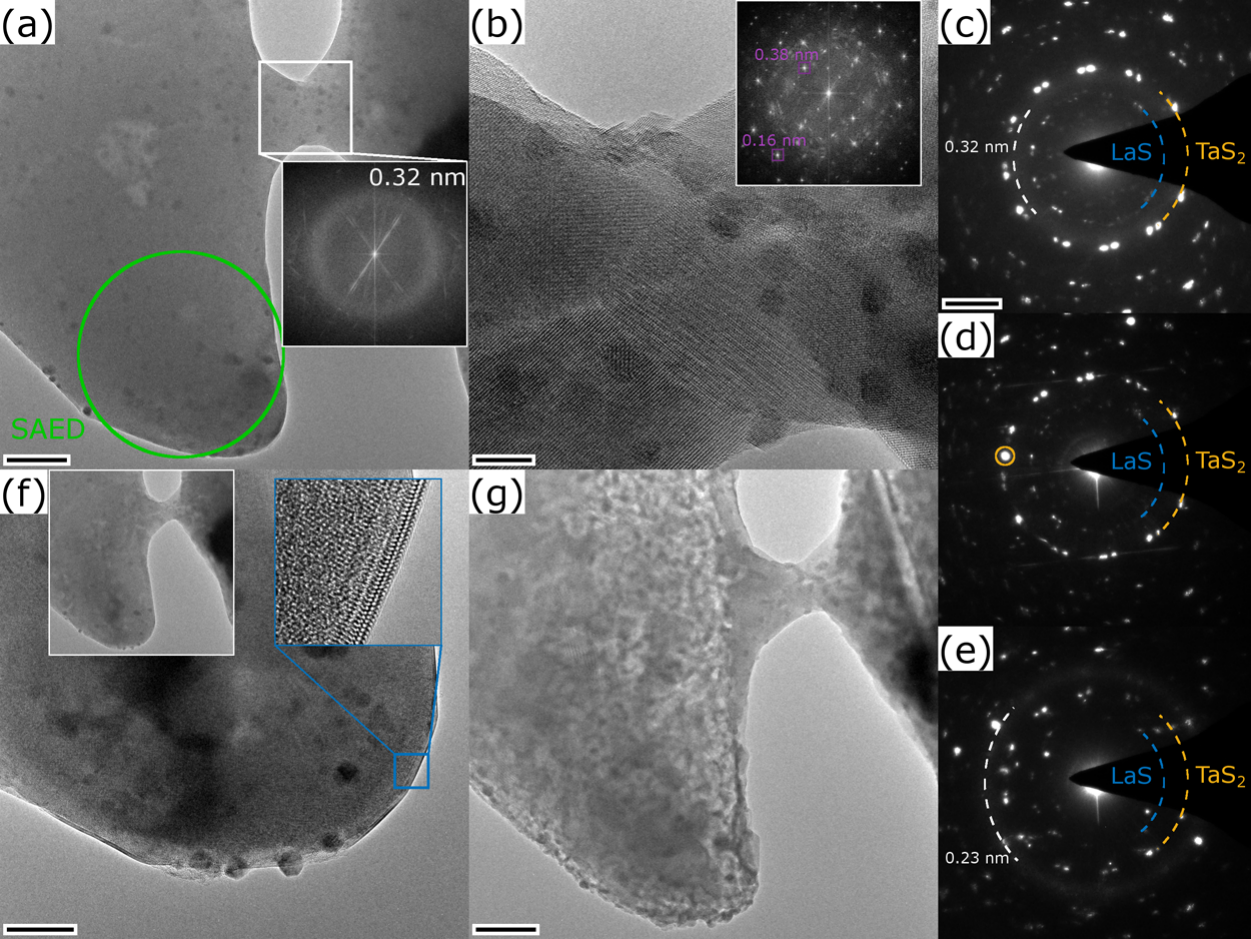
Analysis of the melting process and the starting of the decomposition of the flake during the second in situ experiment. (a) The flake and the bridge appear with homogeneous contrast, the inset power spectrum shows an amorphous phase in the molten bridge. (b) HRTEM image of the bridge that crystallized into several TaS_2_ grains. Two exemplary reflections are marked in the power spectrum in the inset. (c–e) SAED patterns taken from the flake (position marked in (a)) after the melting of the bridge (c), after crystallization into TaS_2_ and starting of the decomposition (d), and after considerable decomposition and formation of metallic Ta (e). (f) TEM and HRTEM images of the edge of the flake after crystallization into TaS_2_ grains (same stage as in (b)). (g) TEM image showing strong decomposition and grainy contrast linked to Ta metal extraction. Scale bars are (a, g) 50 nm, (b) 7 nm, (c–e) 2 nm^–2^ and (f) 20 nm. Inset power spectra are shown up to (a) 0.2 nm and (b) 0.14 nm. The inset TEM image in (f) has a similar size as (a, g) and the inset HRTEM image has a width of 10 nm.

**Figure 8:**
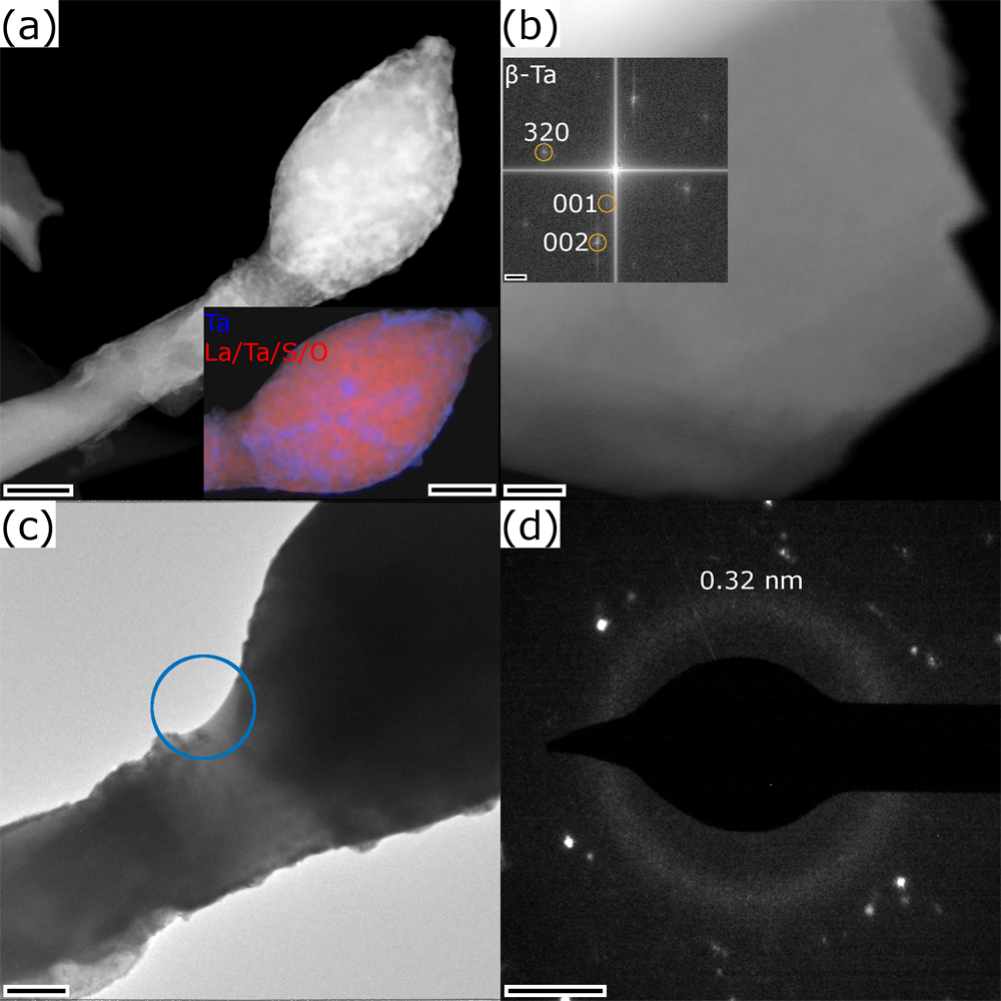
Analysis of the second in situ specimen after breakdown. (a) STEM-HAADF image of the droplet and STEM image in the inset with EDX map overlay separating the two identified phases. (b) STEM-HAADF image of a metallic β-Ta sheet formed at the lower side of the droplet. Three reflection spots are indexed in the power spectrum shown in the inset. (c) TEM image of the interface between the NT and the droplet with position for the SAED acquisition marked. (d) SAED pattern revealing an amorphous ring at 0.32 nm. Scale bars are (a) 200 nm, (b) 4 nm, inset 1 nm^–1^, (c) 90 nm, and (d) 2 nm^–1^.

After several current sweeps up to a maximum of 300 μA, which only yielded minimal structural changes in the specimen, the first stronger change was observed when increasing the current up to 350 μA. Supplementary Video S4 shows the TEM images of the structural evolution during the stepwise sweep. The final state is seen in Figure 6b. When reaching 325 μA, the loosely bound small flake attached to the lower part of the NT (marked in Figure 6a,b), melts and attaches to the close-by flake, forming thereby a conductive bridge. Figure 7a shows a TEM image of the flake and the molten bridge. The molten phase is clearly inferred from the amorphous ring in the power spectrum calculated from a HRTEM image of the bridge area (inset in Figure 7a). It is also confirmed by the SAED pattern taken from the lower part of the MLC flake (Figure 7c), where an amorphous ring (dashed white line) is seen in addition to the reflections from both the TaS_2_ (orange) and the LaS (blue) subsystems. The amorphous ring is centered around 0.32 nm in both the power spectrum and the SAED pattern. Given the elements present in the sample, this amorphous glass phase could be a Ta/La-variant of a S-based metallic glass.^40^ While the electrical current density is thus high enough to melt the small flake, it is still below the threshold to induce a decomposition of both the NT or the flake.

In the following step, a current sweep up to 330 μA was applied. The structural evolution of the specimen during the current sweep was followed by TEM imaging and is shown in Supplementary Video S5. The video reveals the appearance of a grainy contrast in several regions of the flake above a current of 320 μA. Figure 7b,f show HRTEM images of the flake and bridge, revealing that the entire area has fully crystallized. The power spectrum calculated from the image of the bridge area (inset in Figure 7b) shows a large number of reflections. While some could be assigned to either metallic Ta or TaS_2_, a clear assignment to a known structure made of Ta, La, and S could not be established. A different process is observed in the MLC flake, where lattice fringes become visible at its edge, with a parallel orientation with respect to the edge (Figure 7f). In both images, small Pt NPs can be identified, which stem from minor contamination inflicted during the specimen preparation. The SAED pattern acquired from the flake (Figure 7d) reveals four main changes: First, the amorphous ring disappeared; second, an additional, strong [110] reflection of TaS_2_ appeared (marked by an orange circle); third, the intensity of the LaS [110] reflections diminished considerably and finally, slight streaking contrast appears for two sets of TaS_2_ [110] reflections.

These observations can be linked to two independent processes. The loss of the amorphous ring and simultaneous appearance of a TaS_2_ reflection could be explained by the crystallization of a TaS_2_ grain segregated from the amorphous molten material. The second process is related to the MLC flake, where LaS layers start to deintercalate from the edge of the flake leading to the lattice fringes at its edge and the decreasing intensity of the LaS reflections. This deintercalation is going in hand with a slight bending of the flake as visualized from the streaking contrast, typically seen from SAED patterns of NTs (see Figure 1d). This different behavior compared to the extraction of metallic Ta seen in the NT is attributed to the difference between the 1D structure of the NT and the 2D structure of the flake. In contrast to the 1D-NT, the 2D flake possesses open ends, where deintercalation processes are more likely to happen than in the NT, where each layer is closed.

The experiment was continued with repeated applications of electrical current sweeps up to a maximum current of 400 μA. The structural evolution of the specimen during the last sweep as viewed by TEM imaging is given in the Supplementary Video S6. Figures 6c and 7g show TEM images of the specimen after the current sweep. The video and the images show a considerable decomposition of mostly the flake. In analogy to the first specimen, the video shows also that the NT starts to decompose with protrusions appearing on its edge. The SAED pattern acquired from the flake after this current sweep (Figure 7e) confirms the main decomposition process under strong electrical currents to be the deintercalation/decomposition of TaS_2_ from the MLC and its subsequent crystallization into metallic Ta. The SAED pattern reveals the appearance of a ring and a strong reflection located at 0.23 nm, which corresponds well with the [110] reflection of bcc Ta. Simultaneously, the intensity of the reflections linked to both TaS_2_ and LaS further diminish.

Section S8 sheds light on the protrusion formation on the NT during the in situ experiments performed on the second specimen. The analysis clearly shows the formation of a Ta–Pt alloy, grown through the reaction between the deintercalated Ta and Pt contamination inflicted during the specimen preparation. In contrast to the first specimen, the MLC structure in the second specimen remains intact in most of the inner part of the NT. This observation indicates that the critical current density for the breakdown of the TaS_2_ is only exceeded in the outer layers of the NT, which contribute most to the current flow. Additionally, a considerable contribution to the electrical conduction in this specimen could have been provided by the flake. The stronger decomposition observed in the flake compared to the NT suggests that the flake is less stable, which can be attributed to the open ends of the layered structure, closed in the case of the NT.

The final state of the specimen after the in situ experiment is depicted in Figure 8. To reach this state, several current sweeps were conducted, S9 describes three STEM images acquired during these sweeps. The evolution of the specimen during the final current sweep up to a current of 414 μA was followed by STEM imaging shown in the Supplementary Video S7. The video shows that the onset current for the structural modifications is above approximately 390 μA. The final state, given by a droplet formed at the end of the lower part of the NT (Figures 6d and 8a), is reached within less than 10 μs at the end of the sweep, which is indicated by the sudden change of contrast within three pixels of the STEM image. The breakdown is indicated by a small increase in electrical resistance in the electrical measurements.

The formed droplet appears with brighter, grainy contrast compared to the NT in the STEM-HAADF image shown in Figure 8a, indicating a segregation of Ta (element with highest atomic number in the specimen). This is confirmed by an EDX analysis, which shows that the bright areas correspond to Ta, while the rest of the material is a mixture of Ta, La, S, and O (S10). Segregated Ta sheets could be found at the edge of the droplet as revealed by an EDX analysis (S10) and its crystal structure, as β-Ta can be identified using a power spectrum analysis, see Figure 8b. The areas with brighter contrast in the STEM-HAADF image found in the droplet form a sharp border with the NT. An interfacial zone separating the NT and the droplet with decreased and rather homogeneous intensity can be observed (Figure 8a). This zone exhibits an increased intensity in TEM imaging (Figure 6c). An SAED pattern acquired from the edge of this transition area clearly indicates the presence of an amorphous phase as revealed by the broad ring located at 0.32 nm, similar to the molten phase observed in Figure 7a,c. An EDX spectrum taken from that area indicates a composition of La:Ta:S of 67:23:10 at% of the main constituents, but the additional presence of oxygen cannot be excluded (S10).

The comparison of Raman spectra taken from the second specimen on the in situ chip before and after the experiment shows a strong modification of the Raman response (S11). The modes related to TaS_2_ are broadened and their intensity is largely decreased after the current sweep. The appearance of modes at low Raman shifts could be linked to metallic Ta. In contrast, the modes related to the LaS subsystem exhibit only smaller changes, suggesting that the main chemical modifications can be attributed to the decomposition of the TaS_2_ layers.

## Conclusions

We have investigated the breakdown behavior of nanostructures of the misfit layered compound (MLC) LaS-TaS_2_ under extreme electrical currents by in situ transmission electron microscopy (TEM). The experimental results allow to observe the following main physical/chemical processes:

- Breakdown of the TaS_2_ layer in nanotubes (NTs) above current densities of 8.75–10 × 10^5^ Acm^–2^. The breakdown leads to segregation of Ta from the MLC that crystallizes into bcc, fcc, and β-Ta crystal structures.
- Mechanical rupture of the nanostructures upon switching off the electrical current (Joule heating) due to fast quenching to room temperature.
- Removal of La and S from the blend of metallic Ta and remaining LaS_*x*_ material due to Joule heating once the MLC structure has broken down.
- Deintercalation of LaS from the edges of MLC flakes located outside of the main current path and rolling up of the flake.
- Sudden rupture within a few μs of the device made up of a flake and NT resulting in a droplet formation caused by the high electrical currents.
- Generation of an unknown molten glassy phase based on La, Ta, and S.

The experimental results confirm that TaS_2_ is the current-carrying layer in the MLC. They also reveal that a tubular structure is more stable due to the lack of open ends present in flakes. This work illustrates the potential of in situ TEM investigations with high electrical current density. The electrical breakdown and the induced structural and compositional changes during the current sweeps can be tracked with high spatial resolution.

## Supplementary Material

















## Notes

Supplementary video S1, TEM image evolution of specimen 1 under application of an electrical current sweep up to 325 μA (Phase 1) (MP4)
Supplementary video S2, STEM-HAADF image evolution of specimen 1 under application of an electrical sweep current up to 300 μA (Phase 2) (MP4)
Supplementary video S3, STEM-HAADF image evolution of specimen 1 under application of a manually increased electrical current up to 140 μA (Phase 3) (MP4)
Supplementary video S4, TEM image evolution of specimen 2 under application of an electrical current sweep up to 350 μA (MP4)
Supplementary video S5, TEM image evolution of specimen 2 under application of an electrical current sweep up to 330 μA (MP4)
Supplementary video S6, TEM image evolution of specimen 2 under application of an electrical current sweep up to 400 μA (MP4)
Supplementary video S7, TEM image evolution of specimen 2 under application of an electrical current sweep up to 414 μA (MP4)
Additional experimental data including SEM, TEM, and STEM images, EDX data, FFT analysis, and Raman spectra comparison (PDF)

## #

Laboratorium für Elektronenmikroskopie, Karlsruher Institut für Technologie, Karsruhe, Germany
