# Supplementary material for: Unraveling the Decomposition Pathways of LaS-TaS2 Misfit-Layered Compound Nanostructures under Extreme Electrical Currents by In Situ TEM
Source: J Phys Chem C Nanomater Interfaces. 2025 Jul 21;129(30):13803–12. doi: 10.1021/acs.jpcc.5c03498 (PMC12319908; doi:10.1021/acs.jpcc.5c03498)
Supplement: Supplementary file 8 [file jp5c03498_si_008.pdf]

Supplementary information for

Unraveling the Decomposition Pathways of LaS-TaS_2 Misfit-Layered Compound Nanostructures under Extreme Electrical Currents by *in-situ* TEM

Simon Hettler[a,b,*], MB Sreedhara[c], Reshef Tenne[d], Raul Arenal[a,b,e,*]

[a] Instituto de Nanociencia y Materiales de Arag´on (INMA), Universidad de Zaragoza, Zaragoza, Spain
[b] Laboratorio de Microscop´ıas Avanzadas (LMA), Universidad de Zaragoza, Zaragoza, Spain
[c] Solid State and Structural Chemistry Unit, Indian Institute of Science, Bengaluru, 560012 India
[d] Department of Molecular Chemistry and Materials Science, Weizmann Institute of Science, Rehovot 7610001, Israel
[e] ARAID Foundation, Zaragoza, Spain

hettler@unizar.es, arenal@unizar.es

S1: Design of custom *in-situ* chips
S2: Preparation of first *in-situ* specimen
S3: EDX of extracted Ta
S4: FFT analysis of β-Ta
S5: Expanded MLC structure of inner cone
S6: EDX and HRSTEM image after 2nd rupture
S7: Analysis of metallic Ta structure
S8: Formation of Ta/Pt NPs
S9: STEM images of second specimen
S10: EDX spectra of droplet
S11: Raman spectra comparison

Videos:

- SuppVideo_S1: TEM image evolution of specimen #1 under application of an electrical current sweep up to 325 µA (Phase 1). Scale bar is 300 nm.
- SuppVideo_S2: HAADF-STEM image evolution of specimen #1 under application of an electrical sweep current up to 300 µA (Phase 2). Scale bar is 200 nm.
- SuppVideo_S3: HAADF-STEM image evolution of specimen #1 under application of a manually increased electrical current up to 140 µA (Phase 3). Contrast on the right-hand side of the video has been increased to visualize the underlying carbon film. Scale bar is 70 nm.
- SuppVideo_S4: TEM image evolution of specimen #2 under application of an electrical current sweep up to 350 µA. Scale bar is 100 nm.
- SuppVideo_S5: TEM image evolution of specimen #2 under application of an electrical current sweep up to 330 µA. Scale bar is 100 nm.
- SuppVideo_S6: TEM image evolution of specimen #2 under application of an electrical current sweep up to 400 µA. Scale bar is 100 nm.
- SuppVideo_S7: TEM image evolution of specimen #2 under application of an electrical current sweep up to 414 µA. Scale bar is 200 nm.

S1. Design of custom *in-situ* chips

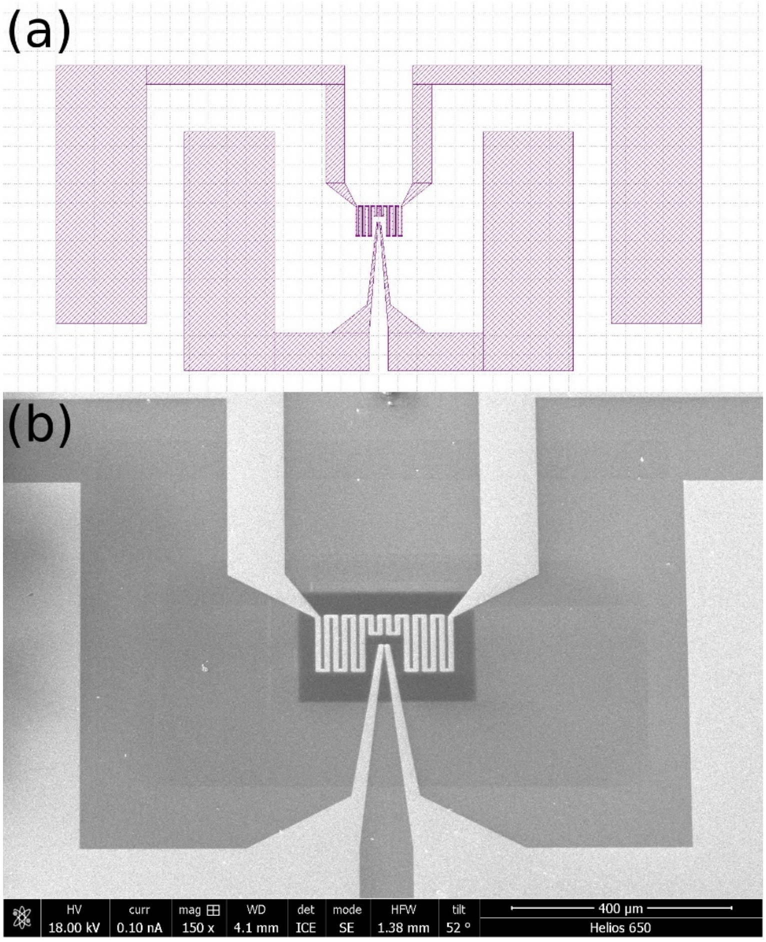

Figure S1: (a) Sketch of the electrode + heating design and (b) SEM image of the heating element and contacts on a microchip with SiNx membrane in the center.

S2. Preparation of first *in-situ* specimen

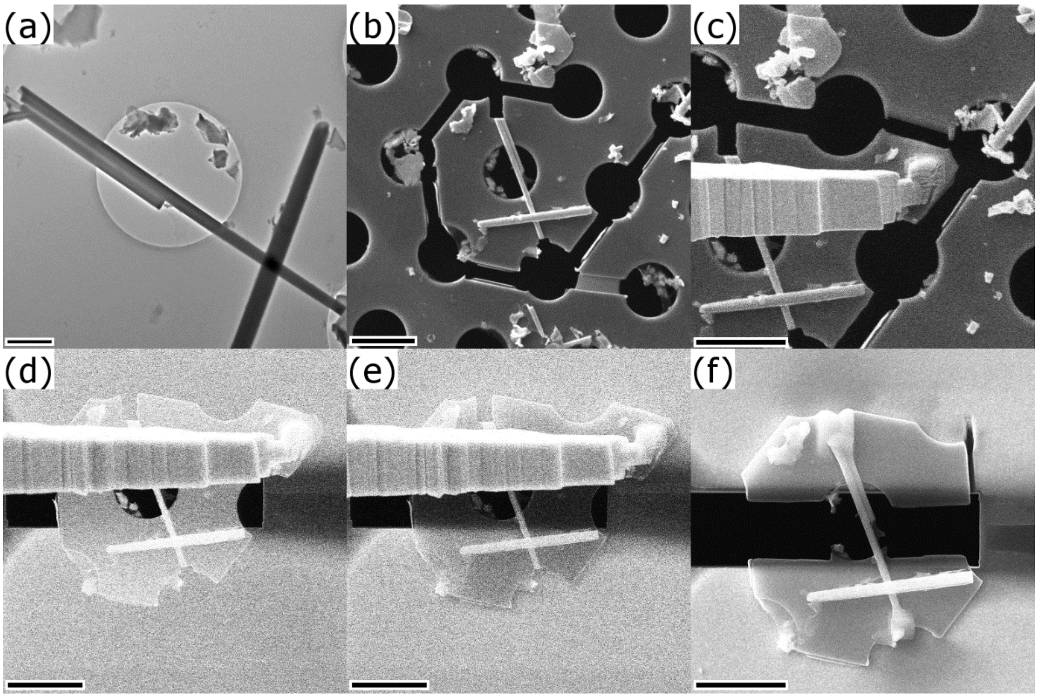

Figure S2.1: (a) TEM image of the bundle of NTs suspended on a SiN membrane. (b) SEM image of the identical position with area cut free. (c) SEM image of the sample with micro-needle contacted to the membrane + nanomaterial. (d) SEM image of the specimen above the *in-situ* chip with hole between the contacts. (e) SEM image of the membrane in contact with the chip. (f) SEM image of the membrane + nanomaterial fixed and electrically contacted by Pt FIBID and with removed SiN membrane in the central part to leave the NTs as sole electric connection. Scale bars are (a) 800 nm and (b-f) 3 µm.

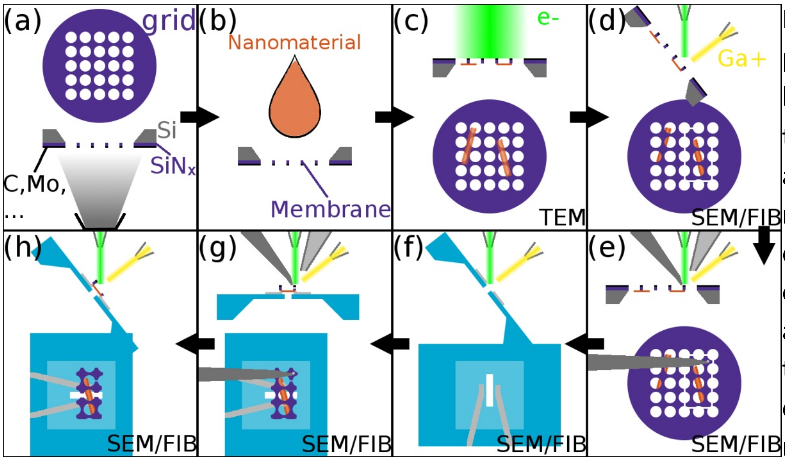

Figure S2.2: Sketch of the transfer process, which starts with a) a holey SiN_x TEM grid coated from the front side with 10 nm of, e.g., amorphous carbon. b) The nanomaterial is subsequently deposited on the back side. c) One or several suitable nanomaterials are selected in a TEM analysis of the sample. d) The membrane containing the selected nanomaterial is cut by FIB at a sample tilt of 52°, leaving a bridge to the surrounding membrane. e) The nanomaterial sustained by the membrane is lifted out using a micro needle and FIBID followed by FIB cutting of the remaining bridge. f) A hole is milled between the contacts of an in situ chip. g) The membrane + nanomaterial is put in contact with the chips' contact pads with the micro needle and a FIBID process. h) The SiN_x membrane in the hole area is removed by FIB, leaving the nanomaterial as only connection between the pads of the chip. Adapted from Hettler et al, *Small Methods* 2024, 8, 2400034. DOI.

S3. EDX of extracted Ta

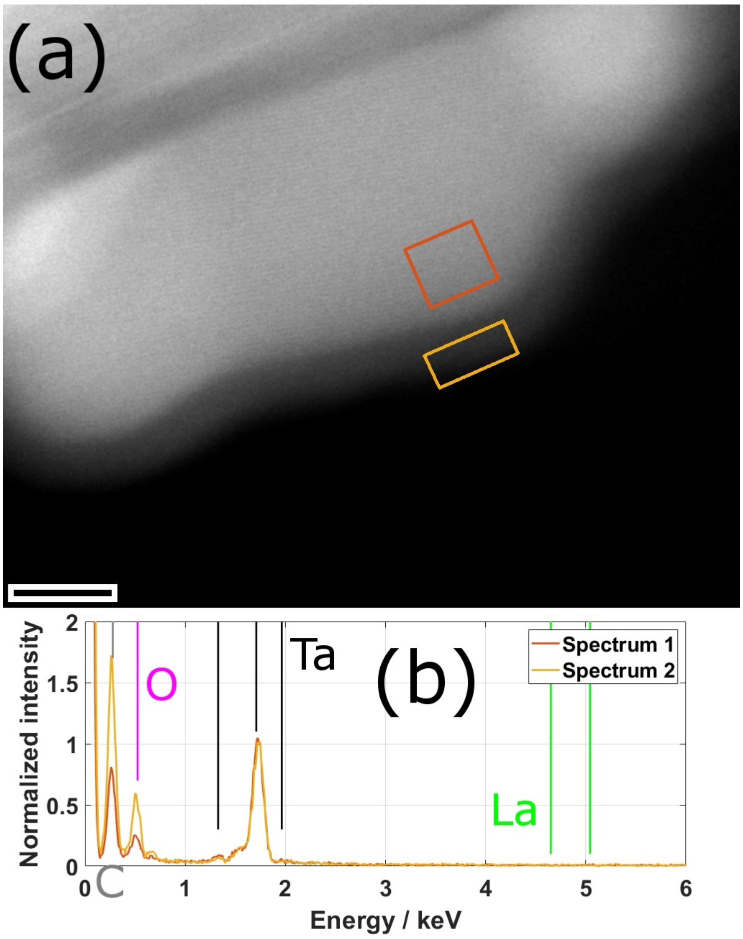

Figure S3: (a) HAADF STEM image of islands grown at the NTs' edge (Figure 2b of main article) with positions of spectra shown in (b) marked. Scale bar is 5 nm. (b) Comparison of two spectra acquired from the island clearly revealing the presence of Ta, O and C (contamination) and the absence of La and S.

S4. FFT analysis of β-Ta

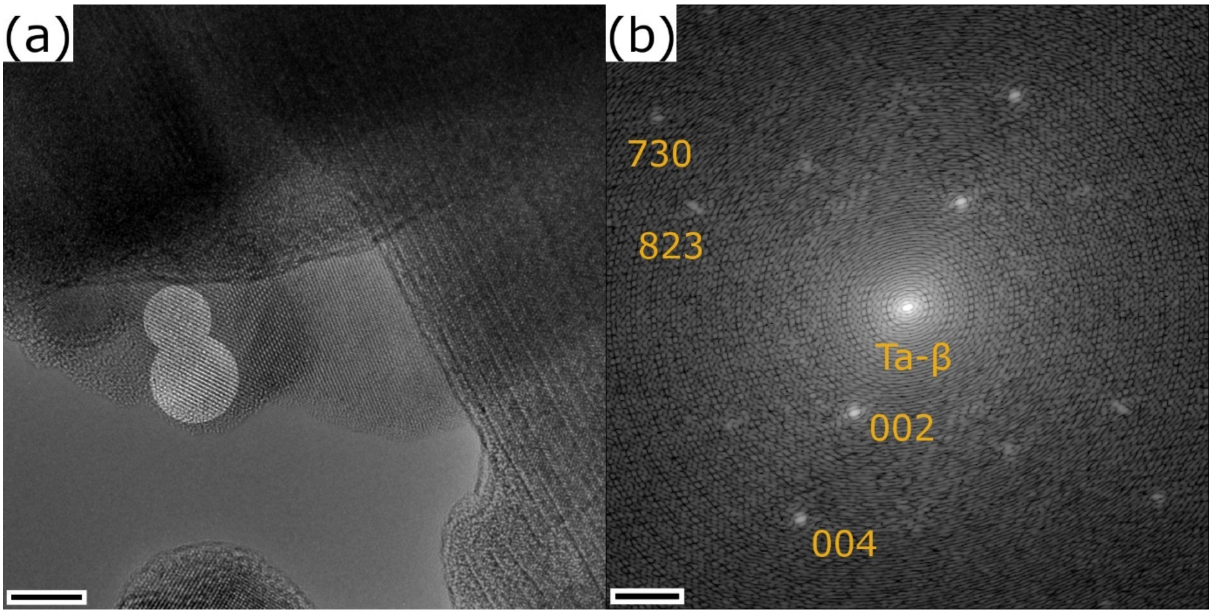

Figure S4.1: (a) HRTEM image with area used for power spectrum calculation highlighted. (b) The spots visible in the power spectrum can be linked to different lattice spacings of β-Ta as indicated.

S5. Expanded MLC structure of inner cone

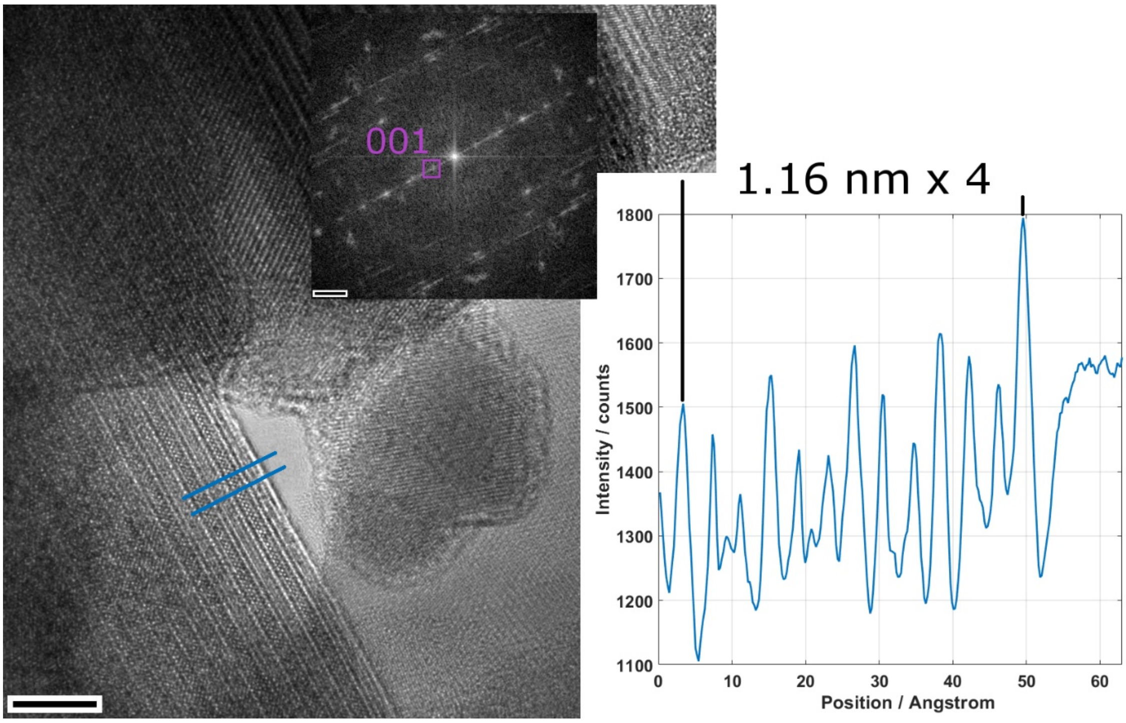

Figure S5: HRTEM image of the ruptured NT with position of line scan marked by blue lines. Inset power spectrum clearly reveals the 001 and corresponding higher orders of the MLC structure. MLC c-axis is observed to be slightly expanded to 1.16 nm instead of nominal 1.15 nm.

S6. EDX and HRSTEM image after 2nd rupture

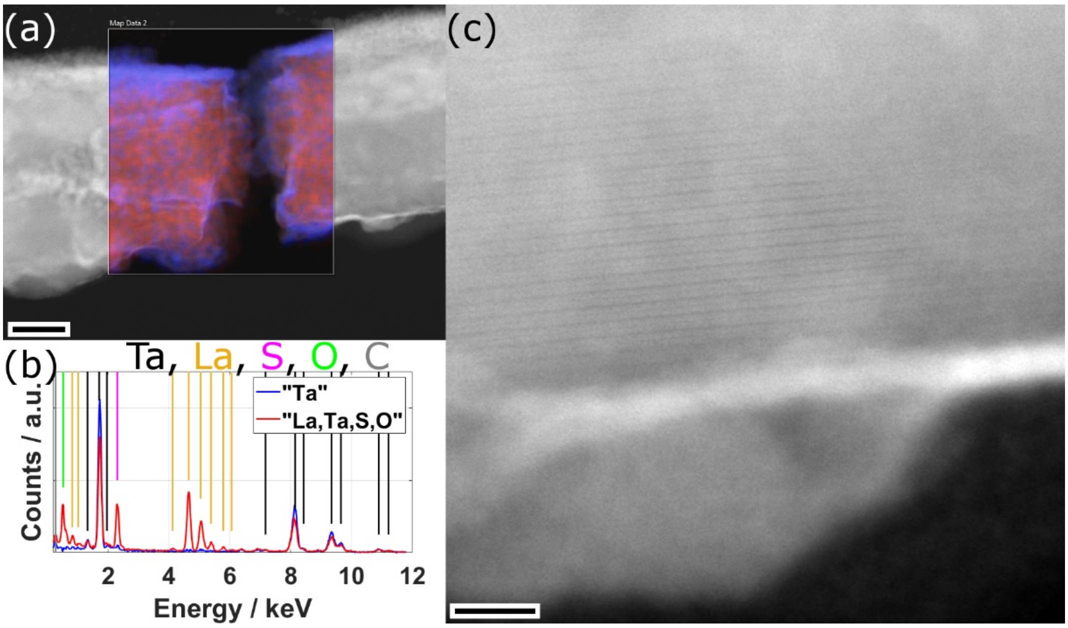

Figure S6: (a) HAADF STEM image with EDX map overlay obtained from a statistical analysis. Blue and red colors correspond to respective spectra in (b). (c) HAADF-STEM image after 2nd rupture showing the Ta soldering as bright line and the double-slab structure of the deintercalated LaS_x similar to the image in Figure 2d in main article. Scale bars are (a) 50 nm and (c) 7 nm.

S7. Analysis of metallic Ta structure

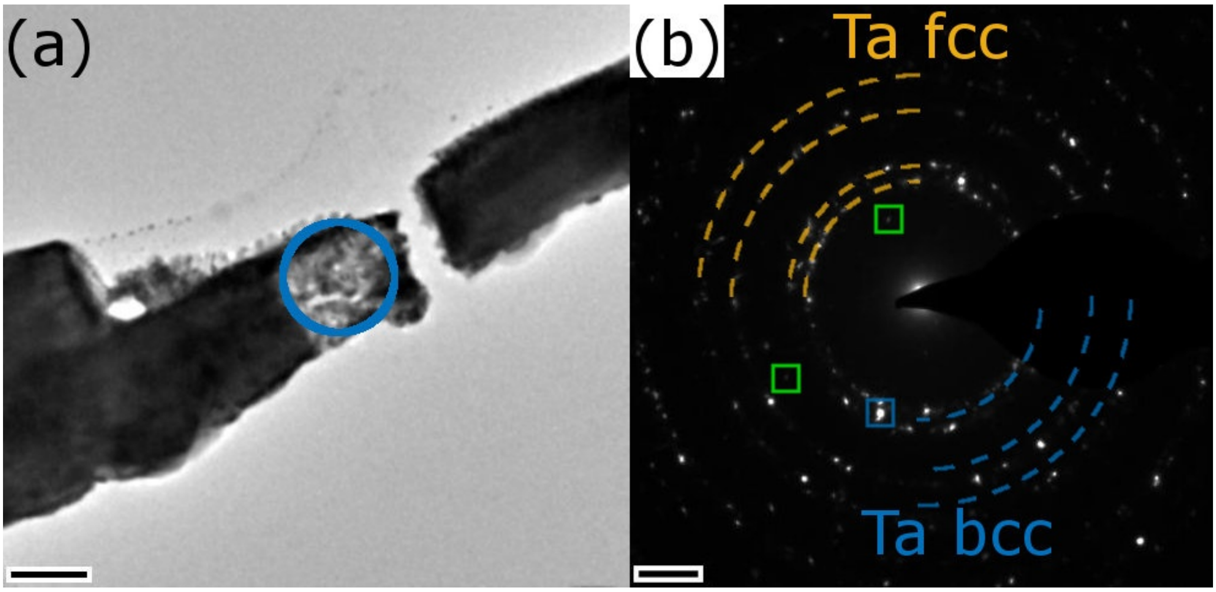

Figure S7: (a) TEM image of final state of the first *in-situ* specimen with position for SAED acquisition marked. (b) SAED pattern taken from the metallic Ta part with the first reflections of Ta fcc and bcc indicated. Most spots coincide with the fcc structure (orange), but one strong reflection is linked to bcc (blue), while others can only be attributed to the β-Ta structure (green).

S8. Formation of Ta/Pt NPs

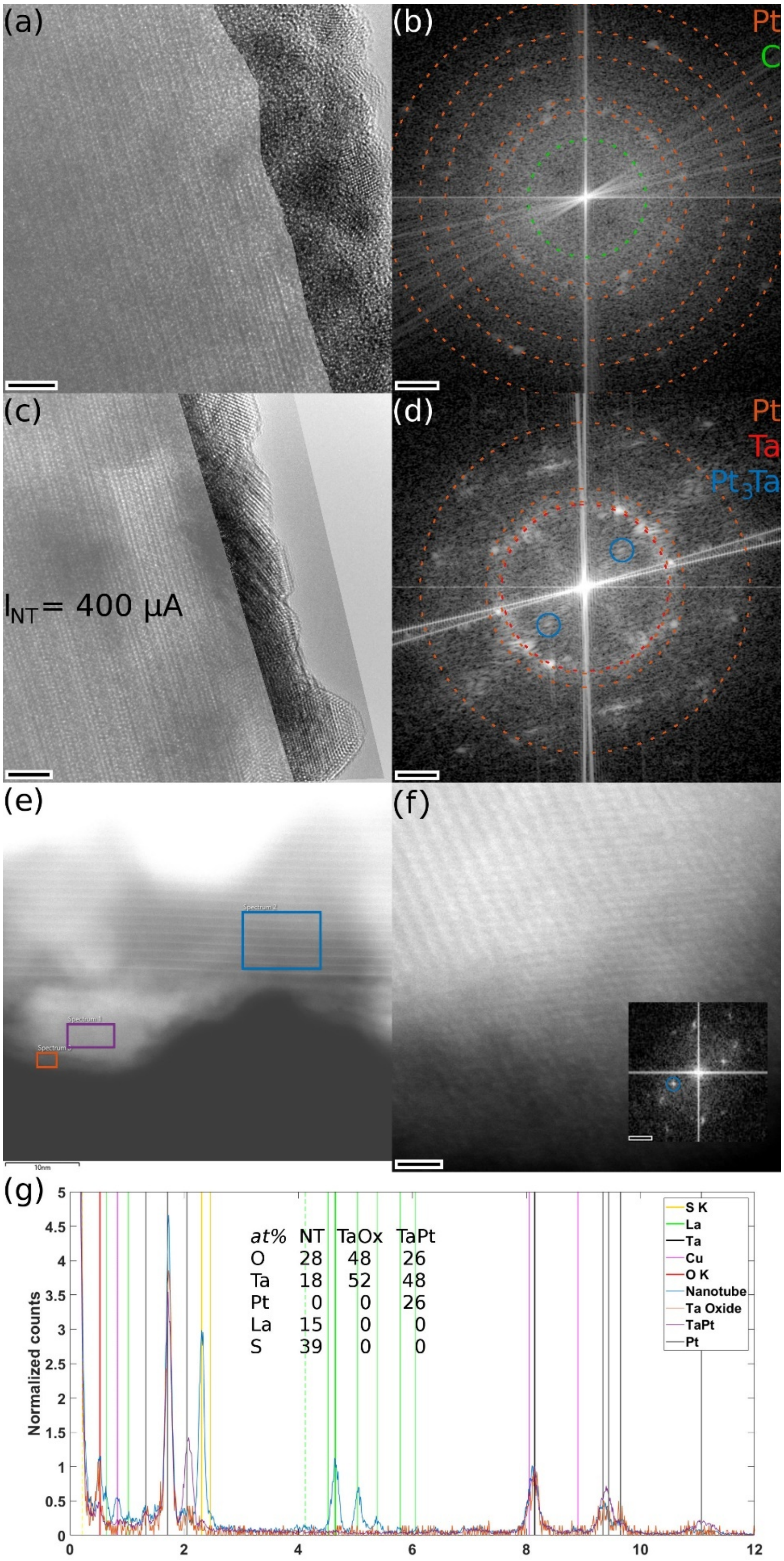

Figure S8: Description see accompanying text. Scale bars are (a,c) 3 nm, (b,d) 2 nm^{-1} and (f) 1 nm / 2 nm^{-1}.

Here we describe the growth of Pt/Ta NPs on the NT surface of the second *in-situ* specimen described in the main article. Initially, Pt NPs form within a C matrix from the inflicted contamination during the preparation process during specimen heating. This is seen from the power spectrum (b) obtained selecting only the surface area of the NT (a). In the power spectrum, an amorphous C ring (green) and several spots linked to Pt fcc can be observed (see table S8.1 for quantitative analysis).

After the sweep up to 400 µA (video S6, Figure 6e and g in main article), the amorphous C has disappeared and highly crystalline NPs can be seen on the NT surface (c). A power spectrum calculated only from the surface area is shown in (d) and the reflections can be linked to both Pt fcc and Ta bcc (orange and red), but also to the Pt_3Ta alloy (COD #1523694, [111] reflection with 0.366 nm marked in (d)). Some spots in the power spectrum stem from the underlying MLC, indicating that the NPs grow directly on the NT.

The presence of a Pt-Ta alloy is confirmed by

EDX.EDX signal has been acquired from a larger NP at the surface (e), and a Ta-Pt NP with oxidized TaO_x surface is found as shown in (g). In the power spectrum of a HRSTEM image (f), a strong reflection is found at 0.37 nm, that does not fit to Pt or Ta, but would again agree with Pt_3Ta. The surface layer appears amorphous and reduces readily under the intense electron beam, making a quantitative determination of the exact alloy and oxide structure difficult.

Pt fcc hkl	COD 9008480 (nm)	Measured (nm)	Deviation (%)
111	0,227	0,226	0,56
200	0,196	0,194	0,83
220	0,139	0,137	1,18
311	0,118	0,117	1,00
222	0,113		
400	0,098	0,097	1,17

Table S8.1: Reflection distances measured for SI8b and compared with Pt fcc (COD: 9008480).

S9.STEM images of second specimen

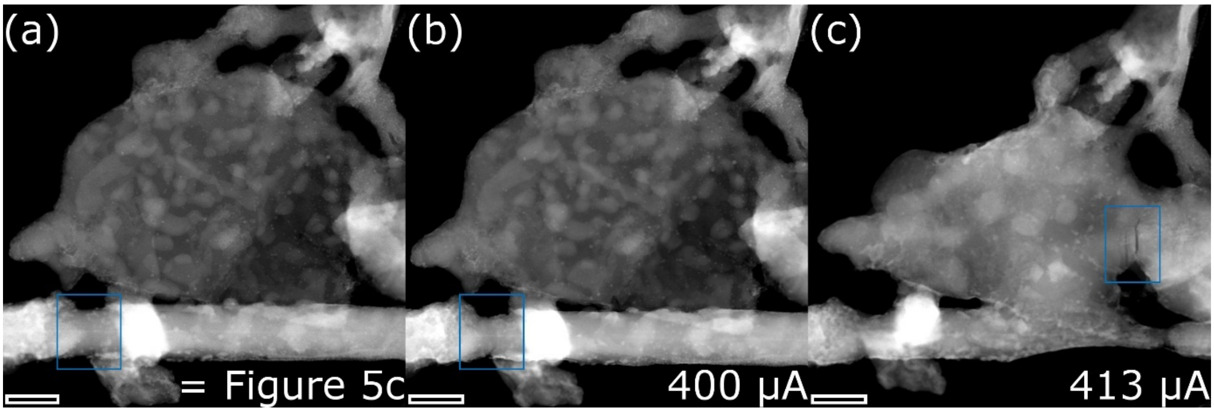

Figure S9: STEM images acquired at different points of the specimen between the states depicted by TEM images in Figure 5c and 5d of the main article. (a) corresponds to Figure 5c but acquired in STEM mode. (b) STEM image acquired after a current sweep up to 400 µA shows a narrowing of the NT mainly on its left side (marked in (a) and (b)). (c) STEM image after a sweep up to 413 µA shows a strong modification: a thinning of the NT on the right, a reduction in size of the flake and a connection of the flake with the NT. On the right side of the flake some dark vertical lines can be seen (marked), which we attribute to mechanical cracking induced by the fast quenching. Scale bars are 200 nm.

S10. EDX spectra of droplet

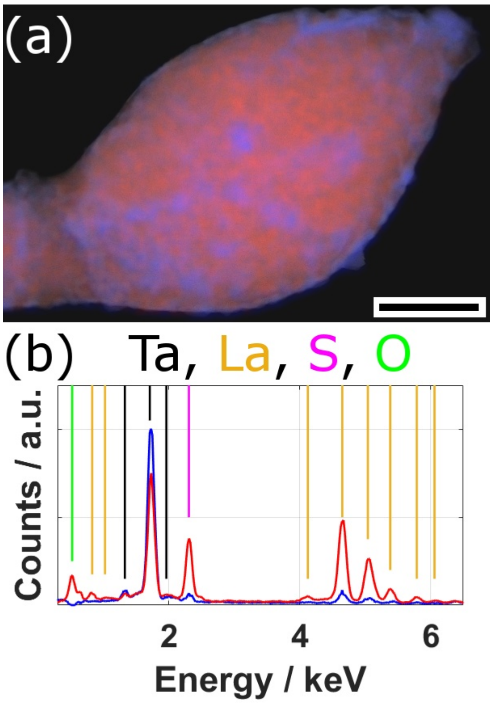

Figure S10.1: EDX analysis of the droplet. The HAADF STEM image in (a) was overlaid with a color map obtained from a statistical analysis of the data set. The blue and red color in (a) corresponds to the blue and red spectrum in (b), respectively. In contrast to the map and spectra shown in Figure 4 in the main article, here the "Ta" spectrum still contains some La,S signal, which we attribute to a fluorescence signal caused by the large size of the droplet. Scale bar in (a) is 200 nm.

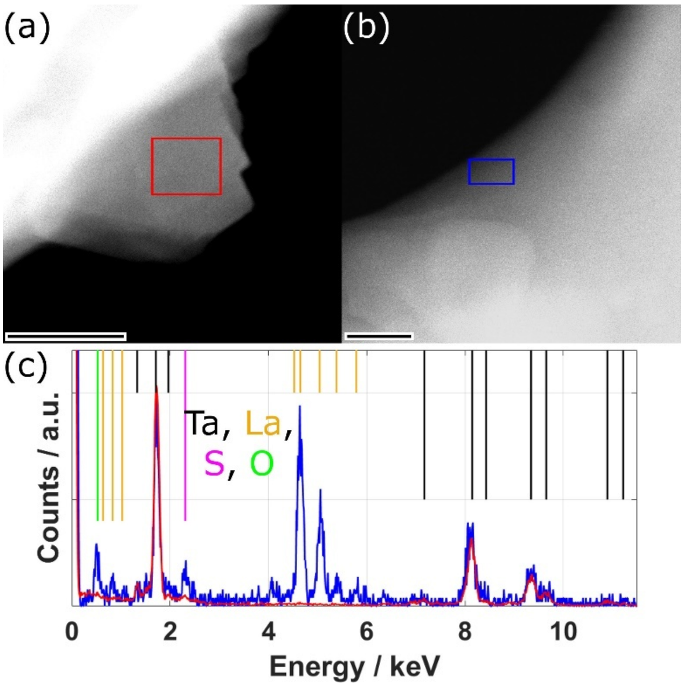

Figure S10.2: Additional EDX analysis of the droplet. A spectrum acquired from the extracted sheet shown in (a) is depicted by a red line in (c) and reveals the presence of only Ta. A spectrum acquired from the amorphous area (b) reveals the presence of all main elements (Ta,La,S) and potentially some Oxygen. Scale bars are (a) 25 nm and (b) 10 nm.

S11. Raman spectra comparison

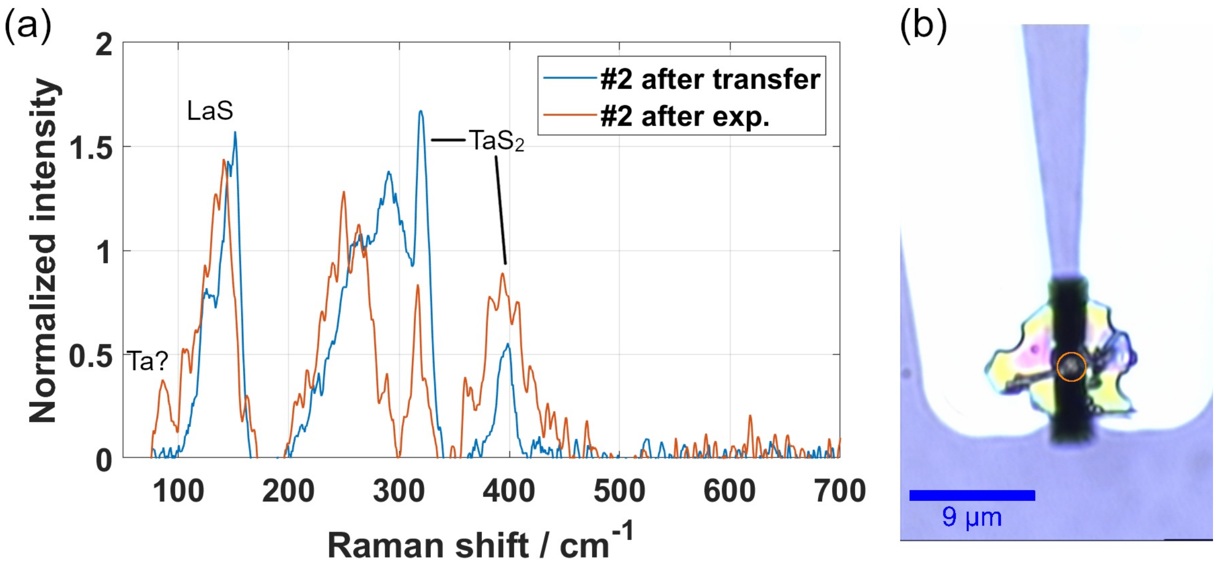

Figure S11: (a) Comparison of Raman spectra taken from the specimen on the *in-situ* chip after transfer (blue curve) and after the *in-situ* experiment (red). The spectrum after transfer agrees well with previous data on LaS-TaS_2 NTs. The intensity in the modes related to TaS_2 are strongly decreased and broadened after the *in-situ* experiment, while the modes stemming from the LaS subsystem only show a reduced modification. An additional peak at low Raman shifts could be attributed to metallic Ta. In sum, the comparison suggests that the main structural changes due to the electrical breakdown are related to the TaS_2 subsystem. (b) Optical microscopy image with approximate Laser beam position marked. The size of the area with structural changes induced during the *in-situ* experiment is similar to the size of the laser beam and contributions to the Raman spectrum after the experiment (red curve in (a)) from unaffected MLC material cannot be excluded.
